# Development of human antibody fragments using antibody phage display for the detection and diagnosis of Venezuelan equine encephalitis virus (VEEV)

**DOI:** 10.1186/1472-6750-8-66

**Published:** 2008-09-02

**Authors:** Martina Inga Kirsch, Birgit Hülseweh, Christoph Nacke, Torsten Rülker, Thomas Schirrmann, Hans-Jürgen Marschall, Michael Hust, Stefan Dübel

**Affiliations:** 1 Abteilung Biotechnologie, Institut für Biochemie und Biotechnologie, Technische Universität Braunschweig, Spielmannstraβe 7, 38106, Braunschweig, Germany; 2 Armed Forces Scientific Institute for Protection Technologies – NBC Protection (WIS), Humboldtstraße 1, 29633, Munster, Germany

## Abstract

**Background:**

Venezuelan equine encephalitis virus (VEEV) belongs to the Alphavirus group. Several species of this family are also pathogenic to humans and are recognized as potential agents of biological warfare and terrorism. The objective of this work was the generation of recombinant antibodies for the detection of VEEV after a potential bioterrorism assault or an natural outbreak of VEEV.

**Results:**

In this work, human anti-VEEV single chain Fragments variable (scFv) were isolated for the first time from a human naïve antibody gene library using optimized selection processes. In total eleven different scFvs were identified and their immunological specificity was assessed. The specific detection of the VEEV strains TC83, H12/93 and 230 by the selected antibody fragments was proved. Active as well as formalin inactivated virus particles were recognized by the selected antibody fragments which could be also used for Western blot analysis of VEEV proteins and immunohistochemistry of VEEV infected cells. The anti-VEEV scFv phage clones did not show any cross-reactivity with Alphavirus species of the Western equine encephalitis virus (WEEV) and Eastern equine encephalitis virus (EEEV) antigenic complex, nor did they react with Chikungunya virus (CHIKV), if they were used as detection reagent.

**Conclusion:**

For the first time, this study describes the selection of antibodies against a human pathogenic virus from a human naïve scFv antibody gene library using complete, active virus particles as antigen. The broad and sensitive applicability of scFv-presenting phage for the immunological detection and diagnosis of Alphavirus species was demonstrated. The selected antibody fragments will improve the fast identification of VEEV in case of a biological warfare or terroristic attack or a natural outbreak.

## Background

Venezuelan equine encephalitis virus (VEEV) belongs to the Alphavirus genus within the Togaviridae family and was first isolated from horses in the end of the 1930s [1,2]. These viruses have a natural transmission cycle between rodents and mosquitos [3]. Millions of horses were affected by this arbovirus with a fatality rate of up to 80% in epidemics in Central and South America [4].

Several species of this family are pathogenic to humans and are recognized as potential biological warfare agent (BWA) [5]. VEEV is classified as Bioterrorism Agent Category B by the center of Disease Control (CDC). Alphaviruses do not only have the potential for illness and transmission, but they can also be produced in large quantities and are moderately easy to disseminate. Furthermore, these virus species have the capacity to cause human epidemics [6-11]. VEEV causes disease symptoms ranging from mild febrile reactions to fatal encephalitic zoonoses. Outcomes are significantly worse for young and elderly patients, with case fatalities ranging from 4 to 35% [12,13]. These viruses are highly infectious as aerosols [14,15] and an intentional release of sufficient quantities as inhalable small-particle aerosol is expected to infect a high percentage of individuals within an area of a least 10,000 km^2 ^[16]. They can replicate in cell culture to very high titers and are relatively stable to environmental influences [17].

For the surveillance of possible bioterrorism targets and endangered populations, rapid detection and diagnosis of VEEV are of crucial importance. In the past, the generation of monoclonal murine antibodies has improved the fast identification of VEEV infections to locate human and equine outbreaks of encephalitis. On the other hand, monospecific diagnostic monoclonal antibodies (mAbs) against VEEV are either hardly available on the market or too expensive for extensive use. In view of these current limitations the generation of specific high affinity recombinant antibodies may significantly improve the current situation and can make the rapid immunological detection widely available.

A promising method to generate recombinant antibodies against human pathogenic viruses like VEEV is the antibody phage display technology. Using antibody phage display, genotype and phenotype of an antibody fragment are linked by fusing the antibody gene fragment to the minor coat protein III gene of the filamentous bacteriophage M13. The resulting antibody fragment::pIII fusion protein is displayed on the surface of the phage particles [18-21]. The most common antibody formats used for this technology are the Fragment antigen binding (Fab) and the single chain Fragment variable (scFv). In comparison to the Fab, that is consisting of the Fragment determining (Fd) of the heavy chain and the light chain linked by a disulphide bond, the scFv simply consists of the variable region of the heavy chain (V_H_) and the variable region of the light chain (V_L_), connected by a short peptide linker [22,23]. The selection of antibody fragments from antibody gene libraries is performed by an *in vitro *selection process [24,25], that is also referred to as "panning".

In this study, we demonstrated the selection of human antibody fragments from a naïve antibody gene library specific for the detection of VEEV. We describe their immunological properties and discuss their possible application of these antibodies for diagnosis and detection of VEEV after a potential bioterrorism assault or natural outbreak of VEEV.

## Results

### Selection of recombinant antibodies against VEEV from a human naïve antibody library

In order to generate antibody fragments reactive to members of the VEE virus serocomplex the human naive scFv antibody gene library HAL4/7 was used. All pannings were performed in a biosafety level 3 laboratory and the vaccine strain, TC83, as a medically important and epizootic Alphavirus species was used as antigen.

**Figure 1:**
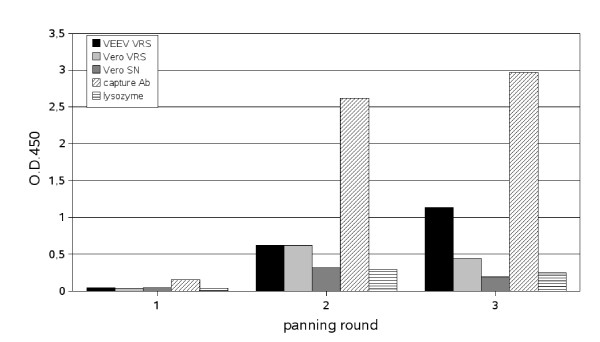
ELISA with 10^10 ^(cfu) polyclonal scFv phage of each panning round. Antigen (directly immobilized): 1 μg VRS purified VEEV particles (VEEV VRS), 1 μg VRS concentrated supernatant from non-infected Vero cells (Vero VRS), 1:2 diluted supernatant of non infected VERO cells (Vero SN), 0.5 μg of each anti-VEEV capture mAb 8747 and VEE-WIS1, 1 μg lysozyme. The bound scFv phage were detected using mAb anti-M13 conjugated with HRP (1:5000).

The phage library was subjected to 3 rounds of panning and representative phage clones were assessed for their ability to bind VEEV TC83 immobilized onto 96 microwell plates. In order to exclude the enrichment of false-positive phage, the binding to supernatant of non-infected Vero cells (VRS concentrated and unconcentrated) was determined. Furthermore, the non-specific bindings of scFv phage to the VEEV-specific capture antibodies mAb 8747 and mAb VEE-WIS without and with an non-specific antigen, like lysozyme, was examined. As shown in figure 1 a significant enrichment of VEEV-specific polyclonal antibody phage occurred after the third panning round. However, besides the specific accumulation of binders also a severe co-enrichment of antibodies to the capture antibodies was observed.

Single clones were isolated from the third panning round. Soluble scFvs were produced in microtitre plates and analyzed by antigen ELISA on immobilized inactivated VEEV particles. The ELISA analysis using soluble scFvs instead of scFv phage minimized the occurance of false positives, because some antibody fragments bind only as antibody phage particles. Inactivated VEEV particles were used to ensure that the antibodies selected on active VEEV particles bound inactivated virus, too. In initial tests, we observed the enrichment of antibodies binding to Vero cell culture components. This effect was enhanced if VEEV particles were directly coated onto the wells and not captured by antibodies. Therefore, VRS purified Vero cell culture supernatant from non virus infected cells was used, because proteins from the cell culture supernatant were also enriched by the VRS system. In total, 230 antibody clones were analyzed by antigen ELISA (data not shown). Due to the signal to noise ratio, 26 VEEV binding scFv clones were further subjected to BstNI fingerprinting to sort out clones with identical restriction pattern (data not shown). After DNA sequencing, 10 different scFvs were finally found from this panning (designated with CHN24-x and MK269-x). One additional scFv clone (MK271-G2) was isolated by a slightly different panning strategy. Interestingly, only antibodies with lambda light chains were obtained. According to the integrative database of germ-line variable genes from the immunoglobulin loci of human (VBASE2) the isolated scFv fragments contained the antigen-binding variable domains of the light chains LV1, 2, 3 and 6. The heavy chains of the isolated scFvs belonged to the subfamily HV1, 3 and 4, while HV1 predominated (table 1).

**Table 1:** Anti-VEEV scFvs

scFv clone	VH			VL	
	
	HV	D	HJ	LV	LJ
CHN24-2-A1	IGHV1-69*01	IGHD2-8*02	IGHJ5*02	IGLV3-1*01	IGLJ3*01
CHN24-2-A2	IGHV3-9*01	IGHD6-19*01	IGHJ3*02	IGLV3-1*01	IGLJ1*01
CHN24-2-B7	IGHV1-18*01	IGHD2-21*02	IGHJ3*02	IGLV2-14*04	IGLJ3*01
CHN24-2-C2	IGHV1-69*01	IGHD6-13*01	IGHJ3*02	IGLV3-21*01	IGLJ3*01
CHN24-2-C3	IGHV3-23*01	IGHD6-13*01	IGHJ6*03	IGLV3-1*01	IGLJ1*01
CHN24-2-D5	IGHV1-8*01	IGHD6-6*01inv	IGHJ6*02	IGLV2-14*04	IGLJ3*02
CHN24-2-F11	IGHV1-18*01	IGHD6-6*01	IGHJ4*02	IGLV6	IGLJ3*02
MK269-C10	IGHV3-30*04	IGHD5-5*01	IGHJ6*02	IGLV2-14*02	IGLJ1*01
MK269-E11	IGHV4-34*01	IGHD3-3*01	IGHJ4*02	IGLV1-51*02	IGLJ3*01
MK269-E12	IGHV4-4*02	IGHD2-21*01inv	IGHJ5*02	IGLV3-21*02	IGLJ3*01
MK271-G2	IGHV1-69*01	IGHD3-16*01	IGHJ6*02	IGLV3-21*02	IGLJ3*02

Tab. 1. Given are the names of the gene segments according to VBASE2. Abbreviations: HV: V (variable) gene segments of the heavy chain; D: D (diversity) gene segment; HJ: J (joining) gene segment of the heavy chain; LV: V gene segment of the light chain; LJ: J gene segment of the light chain.

### Production and characterization of VEEV-specific scFv phage

**Figure 2:**
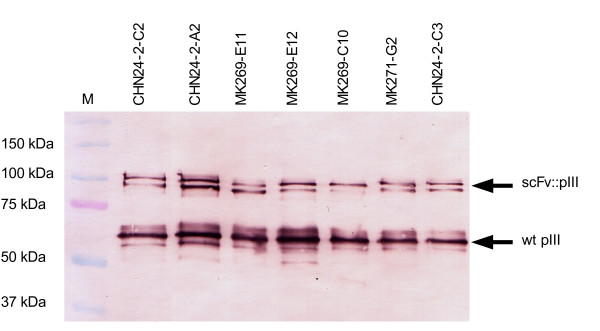
Immunoblot of anti-VEEV scFv phage. 1 × 10^11 ^(cfu) scFv phage per lane were separated on a reducing 10% SDS-PAGE, followed by Western blot and detection of wildtype pIII or scFv::pII fusion using mouse mAb anti-pIII (1:2000) and goat anti-mouse HRP (1:5000). A selection of seven anti-VEEV scFv phage clones is shown.

In order to prove the presentation of functional scFvs on the selected monoclonal scFv phage clones, the clones were subjected to immunoblot analysis. ScFv phage were separated by SDS-PAGE under reducing conditions and the corresponding immunoblot was stained using an anti-pIII mAb. All scFv phage preparations showed a nearly equal and efficient display of scFv antibodies on their surface. This straightly allows to compare the cognate ELISA, Western blot and immunohistochemistry results. The anti-pIII immunoblot of a selection of anti-VEEV scFv phage is shown in figure 2.

### Verification of the VEEV-specific immunoreaction with scFv phage and scFv-Fc fusions

**Figure 3:**
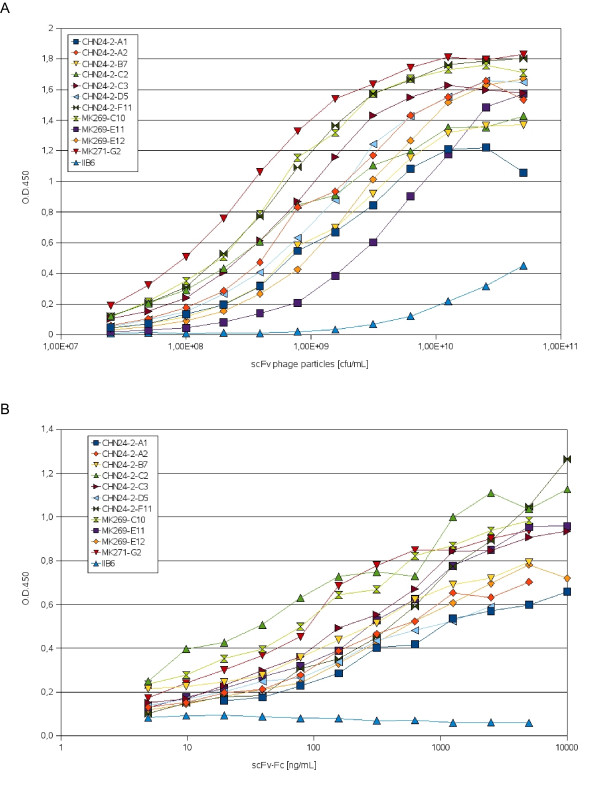
ELISA on directly immobilized inactive VEEV particles. Antigen: 1 μg VRS purified VEEV particles. **A**. A dilutions series of scFv phage particles were used for VEEV detection. The scFv phage were detected using mAb anti-M13 conjugated with HRP (1:5000). The mean values of two ELISAs from two independent scFv phage productions are shown. **B**. A series of scFv-Fc fusion protein dilutions were used for VEEV detection. The scFv-Fc were detected using goat anti-human IgG Fc specific antibody conjugated with HRP (1:20000). IIB6 is a non VEEV-specific control scFv antibody.

**Figure 4:**
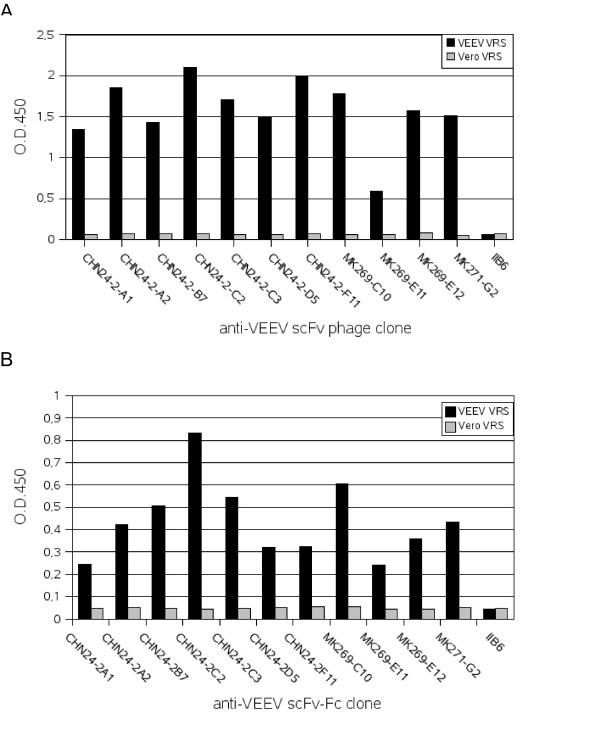
ELISA on directly immobilized active VEEV particles. Antigen: 1 μg VRS purified VEEV particles or 1 μg VRS concentrated supernatant from non-infected Vero cells as control. **A**. 1 × 10^9 ^scFv phage per well were used for VEEV detection. The scFv phage were detected as described in figure 3. The mean values of two ELISAs from two independent scFv phage productions are shown. **B**. 10 ng per well (100 ng/mL) scFv-Fc were used for VEEV detection. The scFv-Fc were detected as described in figure 3.

In order to evaluate whether the antibody format or design influences the specific binding capacity, ELISA results obtained with selected scFv phage (figure 3A) and their corresponding scFv-Fc fusions (figure 3B) were compared. Purified formalin inactivated VEEV TC83 antigen was immobilized onto microwells and serial dilutions of either anti-VEEV scFv phage or serial dilution of scFv-Fc fusion proteins were used for detection. All selected scFv phage clones and the corresponding scFv-Fc fusions were able to bind directly immobilized VEEV particles (figure 3). The background binding of the control antibody IIB6 scFv phage increased when using very high scFv phage particle concentrations. A scFv phage concentration of about 1 × 10^9 ^– 5 × 10^9 ^scFv phage particles, respectively 10–100 ng/mL scFv-Fc fusion proteins are well suited for the detection of immobilized VEEV particles. Additionally, it was also possible to detect direct immobilised active VEEV TC83 particles by ELISA using scFv phage (figure 4A), respectively scFv-Fc fusion proteins (figure 4B).

**Figure 5:**
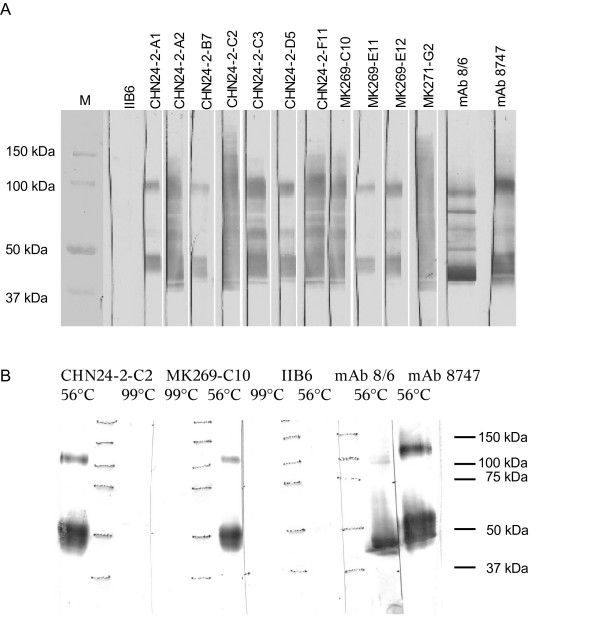
Immunoblot analysis of VEEV antigen detected by scFv phage or scFv-Fc fusions. VEEV VRS preparations were prepared at 56°C under non-reducing conditions and separated by 10% SDS-PAGE. After Western blot membranes were cut in stripes corresponding to 5–6 μg VEEV proteins. **A**. Immunostain was performed with 5 × 10^10 ^(cfu) anti-VEEV scFv phage particles/mL, murine anti-VEEV mAbs 8/6 and 8747 (1:1000) and detected with mAb mouse anti-M13 HRP (1:4000), respectively goat anti-mouse IgG Fc specific HRP (1:10000). IIB6 is a non VEEV-specific control scFv phage. **B**. Additionally, VEEV VRS samples were prepared at 56°C under non-reducing or at 99°C under reducing conditions, respectively. Western blots were stained with 1 μg/mL scFv-Fc and murine anti-VEEV IgG (1:1000) and detected with goat anti-human (gamma chain specific) AP (1:5000), or goat-anti mouse (Fc specific) AP (1:10000), respectively. The marker bands were marked with a pencil.

SDS-PAGE and Western blotting are valuable approaches to examine which VEEV structural proteins are recognized by the selected anti-VEEV antibody fragments. Since the viral glycoproteins E1 and E2 can be separated from each other under non-reducing conditions, virus samples were first disintegrated by incubation for 20 minutes at 56°C in Laemmli sample buffer containing no 2-mercaptoethanol. The samples were separated by 10% SDS-PAGE, blotted onto a PVDF membrane and stained as described. In general 5 × 10^10 ^anti-VEEV scFv phage/mL were used for the specific detection of structural proteins (Fig. 3A). The E2 protein specific antibodies mAb 8747 (Chemicon, CA, USA) and mAb 8/6 (Greiser-Wilke et al., 1989) served as positive control and displayed the expected electrophoretic profile typical for the Alphavirus E2 protein (46,9 kDa) and the cognate viral E1/E2 heterodimer (94,8 kDa). Interestingly, under non reducing conditions most of the anti-VEEV scFv phage displayed a nearly similar binding pattern and were able to bind either the E1 or E2 glycoprotein and the corresponding heterodimer (figure 5A). However, several of the specific scFv phage also caused an undefined smear if used in immunoblot analysis. This might be explained by prolonged staining. If the corresponding scFv-Fc fusions were used for binding, clear and defined bands, representing either E1 or E2 protein, were detectable similar to the E2 glycoprotein positive controls in figure 5B. In contrast, if virus samples were either prepared under reducing conditions or boiled prior to SDS-PAGE, no specific binding was observed. Therefore, the epitopes recognized by the scFv phage and scFv-Fc fusions are likely to be conformation dependent and the secondary structure seems to be critical for the binding of viral structural proteins. None of the isolated scFv fragments identified any linear epitopes.

### Evaluation of the cross-reactivity with different Alphavirus species and subspecies

In order to test the cross-reactivity of the selected antibody clones with other strains of the VEEV as well as with other antigenic complexes, their binding was evaluated in a sandwich antigen catch ELISA by using an Alphavirus specific mAb mixture for capturing and the selected scFv phage for detection.

**Figure 6:**
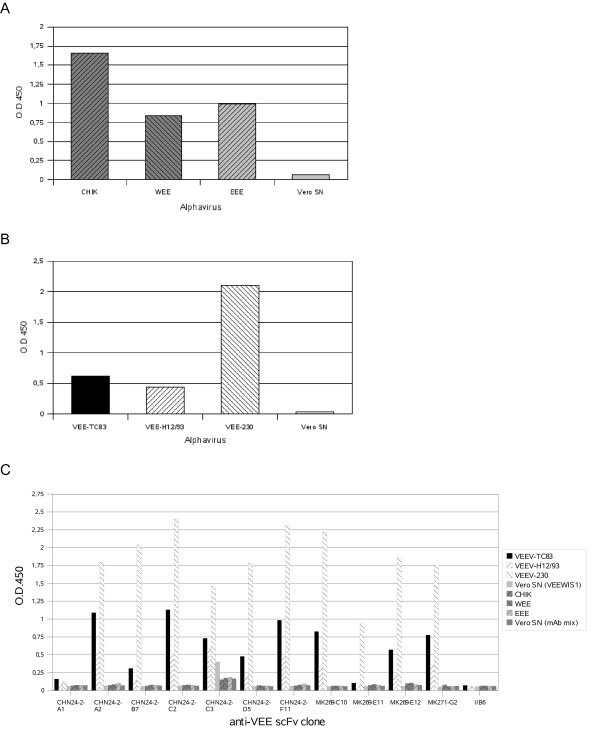
Cross-reactivity of the anti-VEEV scFv clones and different anti-Alphavirus specific mAbs analyzed by ELISA. Antigens: VEEV strains TC83, 230 and H12/93 were captured by using anti-VEEV mAb VEE-WIS1 (3 μg/mL); Eastern equine encephalitis virus (EEE), Western equine encephalitis virus (WEE) and Chikungunya (CHIK) were captured by using an anti-Alphavirus mAb mix consisting of mAb 3/4, mAb 12/2 and mAb VEE-WIS1 (3 μg/mL); Culture supernatant of non-infected Vero cells was captured once by anti-VEEV mAb VEE-WIS1 (VERO VEEWIS1) or by a mAb mix consisting of mAb 3/4, mAb 12/2 and mAb VEE-WIS1 (VERO mAb mix). **A**. Staining with biotinylated anti-VEEV mAb 8/6 (1:10000) and streptavidin conjugated with HRP (1:4000). **B**. Staining with a biotinylated mixture of antibodies consisting of mAb 8/6 (1:10000), mAb VEE-WIS1 (1:10000), mAb 12/2 (1:5000) and mAb 42/2 (1:2000) followed by a streptavidin-HRP (1:4000) incubation. **C**. Staining with 1 × 10^9 ^(cfu) scFv phage per well was followed by an incubation with mAb anti-M13 conjugated with HRP (1:5000). The IIB6 scFv phage was used as negative control. The mean values of two ELISAs from two independent scFv phage productions are shown.

An established VEEV-specific (figure 6A) and Alphavirus genus-specific sandwich ELISA (figure 6B) served as positive control. As negative control, cell culture of non-infected Vero cells was used. As marker antibody the biotinylated anti-VEEV mAb 8/6 was used for the detection of all VEEV strains (figure 6A) and a biotinylated mixture of antibodies consisting of mAb 8/6, mAb VEE-WIS1, mAb 12/2 and mAb 42/2 was used for the group specific detection of Alphaviruses (figure 4B). All viral antigens were captured by either the VEEV-specific mAb VEEV-WIS1 or a mAb mixture of anti-Alphavirus antibodies, consisting of mAb 3/4, mAb 12/2 and mAb VEE-WIS1 (WIS, Munster, Germany). Some virus strains (VEE-230) were captured better than others (VEE-H12/93).

All VEEV antigens were employed with a nearly similar TCID_50_/mL of 3 × 10^8 ^to 1 × 10^9^. In addition to the VEEV vaccine strain TC83 of subtype IAB, the USSR (Russian) vaccine strain VEEV 230 and the British NCPV strain VEEV 12/93 were applied. Furthermore, the selected scFv clones were tested for the specific detection of Eastern equine encephalitis virus (EEEV) strain H178/99, Western equine encephalitis virus (WEEV) strain H160/99 and Chikungunya virus (CHIKV) strain S27 Petersfield.

Positive ELISA signals were obtained for the different VEEV strains with all tested scFv phage clones (figure 6C). In contrast, the scFv CHN-24-2A1 showed a comparable low antigen binding. Maximum binding in the antigen sandwich ELISA was found for the Russian strain VEEV 230. This might be explained by the fact that this virus sample was chemically inactivated prior to use. We suppose that dependent on the inactivation the critical epitopes are more accessible for antibody detection.

However, none of the selected anti-VEEV scFv phage showed any cross-reactivity with other Alphaviruses, when used as detection antibody. All positive controls exhibited the expected binding pattern and were captured and detected by their specific mAbs in the ELISA.

### Detection of VEEV antigen in lysates of infected Vero cells

In order to examine the broad immunological applicability of the selected scFvs, we also tested the recombinant antibody fragments for the specific detection of VEEV TC83 in lysates of infected Vero cells. These cell lysates were prepared by disrupting infected cells with 4 M urea while coupled to microwells. Detection was performed with scFv phage followed by an incubation with mAb anti-M13 conjugated to HRP. Lysates of non-infected Vero cells and VEEV antigen incubated with the mAb II-B6 served as negative control.

**Figure 7:**
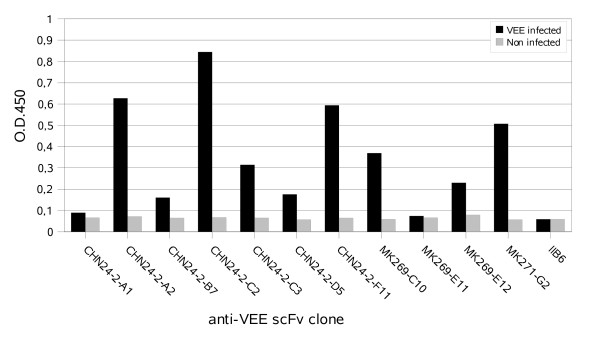
ELISA on VEEV infected cell lysate. Antigen: cell lysate from VEEV infected/non-infected Vero cells. VEEV was detected by using 1 × 10^9 ^(cfu) scFv phage per well followed by an incubation with mAb anti-M13 conjugated with HRP (1:5000). The IIB6 scFv phage was used as negative control. The mean values of two ELISAs from two independent scFv phage productions are shown.

Specific binding could be confirmed for nearly all selected antibody fragments except for the clones CHN24-2-A1 and MK269-E11. The most stringent binding results were obtained with the scFv clones CHN24-2-A2, CHN24-2-C3, CHN24-2-F11 and MK271-G2 (Fig. 7).

In addition, detection of VEEV-specific antigen by immunohistochemistry in TC83 infected and formaldehyde fixed Vero cells was possible. Similar to the results described above, all scFv clones, except for clone CHN24-2-A1, showed a specific cytoplasmic immunostaining of VEEV infected Vero cells (data not shown).

## Discussion

For the detection of VEEV after an potential bioterrorims assault, e.g. by use of a VEEV aerosol, or a natural outbreak of VEEV, a fast diagnosis of these pathogen is necessary. The present work describes for the first time the screening and isolation of anti-VEEV antibody fragments from a human naïve antibody gene library by phage display. Ten out of eleven scFv clones were selected by the panning strategy using a mAb mixture for virus capturing as described. One further clone, MK271-G2, was isolated by an alternative panning, that was performed on directly immobilized viral antigen. The use of antibody captured virus particles was the preferred panning strategy because preliminary tests revealed, that panning on directly immobilized VEEV antigen enhanced especially the enrichment of non-specific binders.

To our knowledge, there are no studies demonstrating the successful *in vitro *antibody selection against human pathogen complete virus particles using naïve antibody gene libraries. A successful panning against severe acute respiratory syndrome coronavirus using a human semisynthetic library is described by van den Brink et al. [26]. In most other studies recombinant or purified virus proteins were used for panning if using a naïve antibody gene library [27-29]. The pannings using complete particles are mostly performed using immune libraries, e.g de Carvalho et al. [30], Koch et al. [31] or Duan et al. [32]. The panning procedure described here might be also useful for the *in vitro *antibody selection of scFvs against other viral targets from human naïve antibody gene libraries, in particular when either immunized patients are not available or immunisation is not ethically feasible.

Nearly all scFvs were able to detect active as well as inactive VEEV TC83 viral antigen. Comparable indirect ELISA data were obtained with scFv phage and their corresponding scFv-Fc fusions. The specific immunoreaction could be verified by Western blot analysis, immunohistochemistry and by immunostaining of urea disrupted cell lysates. The selected antibody clones were reactive with all tested members of the VEE virus serocomplex but showed no significant cross-reactivity with closely related Alphavirus species like WEEV, EEEV and CHIKV, if used as detection molecules.

All Alphaviruses share a number of structural, sequential and functional similarities. Immunological typing approaches categorize the nearly 30 species into seven serocomplexes or species. The nucleotide and amino acid identity among these antigenic complexes, subtypes and varieties varies from 45 to 96 % [2,3,33,34]. In general, the sequences of structural proteins are more divergent than the sequences of non-structural proteins. In immunoblot analysis the anti-VEEV scFv phage displayed a similar binding pattern like the E2 protein specific antibodies mAb 8747 (Chemicon, CA, USA) and mAb 8/6 [35] and identified probably the E2 glycoprotein and the cognate viral E1/E2 heterodimer. Interestingly, all obtained scFv clones identified structural epitopes that are still folded after denaturation at 56°C under non-reducing conditions. In contrast, if the viral antigens were either prepared under reducing conditions or boiled prior to SDS-PAGE, no specific binding was observed.

A possible neutralisation activity of the selected scFvs has to be assessed in further studies. Normally, the protective immunity to Alphaviruses is associated with an antibody reactivity to the virion glycoproteins E2 and so far, six conformationally stable epitopes were identified as critical for virus neutralization [36-40]. Furthermore our antibodies are fully human and therefore better suited for applications like as passive vaccination than murine antibodies.

To date, VEEV diagnosis is performed using monoclonal and polyclonal antibodies [41] and also scFv fragments have been analysed [42]. This study showed that scFv phage are applicable for a broad range of anti-VEEV diagnosis assays: antigen ELISA on purified virus particles, ELISA on cell lysate and immunoblot. Furthermore, the recombinant fragments offer the possibility to develop a VEEV-specific diagnosis assay since the specific scFv phage can be easily produced and purified in high amounts. This could be an alternative to fullsize IgGs for an ELISA assay. At least, they might be applied for immuno-PCR [43] in order to increase the sensitivity of detection. These methods can be used for the diagnosis of VEEV in the enviroment and for the detection of human or equine VEEV infections.

## Conclusion

For the first time, this study describes the selection of antibodies against a human pathogenic virus from a human naïve scFv antibody gene library using complete, active virus particles as antigen. The described antibody selection procedure may also be useful for the *in vitro *antibody selection of antibody fragments against other viral targets from human naïve antibody gene libraries, in particular when immunized patients are not available or immunisation is not ethically feasible. The broad and sensitive applicability of anti-VEEV scFv-presenting phage for the immunological detection and diagnosis of Alphavirus species was demonstrated. The selected recombinant antibody fragments will improve the rapid and specific detection of VEEV infections after human and equine outbreaks of encephalitis, where an early and definite identification is of critical importance.

## Methods

### Cell culture and virus production

Alphaviruses were grown in Vero cells (VERO-B4, African green monkey kidney cells, DSMZ-ACC 33, Deutsche Sammlung von Mikroorganismen und Zellkulturen GmbH, Braunschweig, Germany) in biosafety level 2 and 3 facilities according to standard procedures [35]. Virus titers were determined by the 50 % tissue culture infective dose (TCID_50_/mL) method [44,45]. All viruses used in this study represent models for biowarfare agent relevant Alphavirus species and are either part of the strain collection of the Armed Forces Scientific Institute for Protection Technologies – NBC Protection (WIS) or were received from the National Collection of Pathogenic Viruses (NCPV), UK. The viruses used in this study were VEEV strain TC83 (variety 1AB), VEEV strain 12/93, Eastern equine encephalitis virus (EEEV) strain H178/99, Western equine encephalitis virus (WEEV) strain H160/99 and Chikungunya virus (CHIKV) strain S27. The strain TC83 was obtained from the Trinidad donkey strain by serial passages on guinea pig embryo heart cells in 1960 [46]. Additionally, VEEV strain 230 antigen (inactivated by β-propiolactone) was purchased from Senova GmbH (Jena, Germany). Strain VEEV 230 is the former USSR vaccine strain. Its history of production is not exactly known but it has been produced by serial passages of a virulent natural strain on chick embryos [47]. If not particularily indicated, active virus material was used throughout the study. Lysates of VEEV infected cells were prepared by incubation with 4 M urea for 15 min at room temperature (RT).

### Purification of Alphaviruses

Virus containing supernatants from infected Vero cells were either purified by affinity chromatography on Matrex Cellufine Sulfate Medium™ (*Virus Recovery System*, VRS, Chisso America Inc., NY, USA) or by isopycnic density gradient centrifugation. Matrex Cellufine Sulfate Medium™ (VRS) is a cellulose bead medium functionalized with a low concentration of sulfate esters that operates similar to a cation-exchange resin and has a high affinity to enveloped viruses. It selectively adsorbs complete virus particles as well as viral coats according to their charge. Briefly, 50 mL resin was equilibrated with adsorption buffer (0.01 M phosphate buffer, pH 7.5). Up to 200 mL of virus containing prefiltered cell culture supernatant was loaded onto the column which then was washed twice with 0.01 M phosphate buffer, pH 7.5. Elution of virus particles was performed with 1 M NaCl.

Virus particles were pre-purified using ultracentrifugation through the sucrose cushion method (20% sucrose cushion), which causes low mechanical stress and allows the concentration and collection of morphologically intact particles after centrifugation at 112,000 × g for 2 to 3 hours. The pellet was resuspended in 0.5 to 1 mL phosphate buffered saline (PBS; [48]) and further purified by isopycnic density gradient centrifugation (20 to 60 % sucrose) for 18 hours at 217,500 × g. The virus containing fraction was removed, stored at -80°C until subjected to further analysis.

### Selection of recombinant antibodies

The panning procedure based on protocols by Hust et al. [25] with numerous modifications in 96 well microtitre plates (Maxisorb, Nunc, Wiesbaden, Germany). The mAb 8747 (Chemicon, Temecula, USA; [42]) and mAb VEE-WIS1 (WIS, Munster, Germany) were incubated in concentrations of 1,5 μg/mL each overnight at 4°C in microtitre wells, followed by blocking with 1% (w/v) BSA in PBST (phosphate buffered saline + 1% Tween 20; [48]) for 1 h at RT. For every panning round one well was coated for the selection and one well was coated for a preselection step. For the preselection step 50 μL VRS concentrated supernatant from non-infected Vero cells + 50 μL 1% BSA in PBST was incubated. Afterwards, the wells were blocked with 2% skim milk powder in PBST. After 2 h at RT the wells were washed three times with PBST. In parallel, for selection, 50 μL VRS purified VEEV (2.7 mg/mL) + 50 μL 1% BSA in PBST was captured for 1 h by gently shaking at RT, followed by overnight incubation at 4°C.

The human naïve HAL4/7 antibody gene library [49] consisting of in 5 × 10^9 ^independent clones in total based on the phagemid vector pHAL14 [49,50] was used for panning. The library was packaged using Hyperphage [51-53]. Prior to panning 5 × 10^11 ^scFv phage particles of HAL4 (kappa V_L _repertoire) and 5 × 10^11 ^scFv phage particles of HAL7 (lambda V_L _repertoire) were mixed with 150 μL „panningblock" solution (1% (w/v) BSA +1% (w/v) skim milk in PBST). In the preselection step, the library phage suspension was incubated at RT for 2.5 h in the well with captured VRS concentrated supernatant from non-infected Vero cells to remove non-specific binders. The supernatant containing the depleted library was mixed with 1/10 volume of VRS concentrated supernatant from non-infected Vero cells and 5 μg of a non VEEV-specific murine IgG for competition. For the selection step, the library solution was incubated in the wells with the immobilised VEEV at RT for 2 h followed by 30 times washing with PBST. Afterwards the bound scFv phage particles were eluted with 200 μL trypsin solution (10 μg/mL trypsin in PBS) at 37°C for 30 min. The supernatant containing the eluted scFv phage was transferred into a new tube. For the inactivation of the VEEV particles, 100 μL of 0.1 M glycin buffer pH 2.2 were added and incubated at RT for 15 min. The solution was neutralized with 100 μL 0.1 M phosphate buffer pH 7.6. 10 μL of eluted scFv phage were used for titration as described by Hust et al. [25]. The remaining scFv phage were amplified as described by Hust et al. [25] and used for the next panning round. The second panning round using the amplified phage was performed with the following modifications: the amount of antigen was reduced by 50% and washing cycles during panning were increased to 60. Additionally, in the third panning round the amount of antibody phage was reduced to 1 × 10^9 ^scFv phage. Furthermore, cell culture supernatant from non-infected Vero cells, 1/10 volume, was used for competition.

### Antigen ELISA using scFv phage, scFvs and scFv-Fc fusions

All ELISAs were performed in 96 microtitre well plates (Maxisorb™, Nunc) that were coated with VRS purified viral antigen overnight at 4°C. Afterwards the wells were washed three times with PBST and blocked with 2% (w/v) skim milk powder in PBST (M-PBST) or with 1% fetal calf serum (FCS) in PBST for 1.5 h at RT, followed by three washes with PBST. ScFv phage, soluble antibody fragments or scFv-Fc fusion proteins were diluted in 100 μL M-PBST and incubated with the antigen for 1.5 h, followed by five washes with PBST. Bound scFv phage were detected by using the mAb anti-M13 conjugated with horseradish peroxidase (HRP) (GE Healthcare, München, Germany; 1:5.000). Bound soluble antibody fragments were detected by using the murine mAb 9E10 which recognizes the c-terminal c-myc tag. Staining was performed with a goat anti-mouse Ab conjugated to HRP (Sigma; 1:10.000). The specific binding of scFv-Fc fusion proteins to viral antigen was assessed with a goat anti-human Fc specific mAb conjugated to HRP (Sigma; 1:20.000), biotinylated mAbs were detected by using a Streptavidin HRP conjugate (GE Healthcare; 1:4.000). The visualization was performed with TMB (3,3',5,5'-tetramethylbenzidine) as substrate and the staining reaction was stopped by adding 100 μl 1 M sulphuric acid. Absorbance at 450 nm was measured by using a SUNRISE™ microtiter plate reader (Tecan, Crailsheim, Germany).

### Production of soluble antibody fragments in microtitre plate wells

Microtitre plate wells containing 200 μL 2xTY + 100 mM glucose + 100 μg/mL ampicillin (2xTY-GA) were inoculated with single *E. coli *colonies from the phage titration of the panning and incubated overnight at 37°C and with constant shaking at 1200 rpm. 200 μL 2xTY-GA was inoculated with 10 μL of the overnight culture and grown at 37°C and 1200 rpm for 2 h. Bacteria were harvested by centrifugation for 10 min at 3220 × g. The pellets were resuspended in 200 μl 2xTY + 100 μg/mL Ampicillin + 50 μM isopropyl-beta-D-thiogalacto-pyranoside (IPTG), a substance that induces the prokaryotic *lacZ *promotor in *E. coli*, and incubated at 30°C and 1200 rpm overnight. Cells were removed from the scFv containing supernatant by centrifugation for 10 min at 3220 × g and 4°C.

### Sequencing

Sequencing was performed using ABI Prism 310 Genetic Analyzer according to the manufacturers instructions using oligonucleotide primer MKpelB_f (5' GCCTACGGCAGCCGCTGG 3') or MKmyc_r (5' GATCCTCTTCTGAGATGAG 3'). The antibody gene fragments were analyzed by using VBASE2 [54,55].

### SDS-PAGE and immunoblot analysis

In order to analyse the scFv presentation on phage, the SDS-PAGE (sodium dodecyl sulphate-polyacrylamid gel electrophoresis) of scFv phage followed by Western blot and immunostaining of pIII were performed as described by Kirsch et al. [56].

ScFv-Fc fusion proteins and scFv phage were used to detect VEEV proteins. VEEV particles were separated by SDS-PAGE and blotted onto PVDF membrane. The membrane was blocked with M-PBST for 1 h at RT. ScFv phage or the scFv-Fc fusion protein were incubated for 1.5 h at RT, followed by two times washing with PBST. For the detection of bound scFv phage mAb mouse anti-M13 conjugated with HRP (GE Healthcare, 1:4000) was used for detection and visualized by DAB (diaminobenzidine). For the detection of the scFv-Fc fusion protein goat anti-human (Fc specific) conjugated with alcaline phosphatase (AP) (Dianova, Hamburg, Germany, 1:5000) was used. Murine IgGs were detected using goat anti-mouse (Fc specific) conjugated with AP (Sigma, 1:10000) and visualised by NBT/BCIP.

### Cloning and production of scFv-Fc fusion proteins

VEEV-specific scFv gene fragments were subcloned from the library vector pHAL14 into the mammalian expression vector pCMV-hIgG1-Fc-XP (Schirrmann, manuscript in preparation) between the murine IgG signal peptide and the human IgG1 gene fragments by using the restriction sites *Nco*I and *Not*I. For the transient production of VEEV-specific scFv-Fc fusion proteins, the human embryonic kidney (HEK) cell line, 293T (American Type Culture Collection, ATCC, Rockwell, MD, No. CRL-11268) was transiently transfected by using the cell line specific lipid transfection reagent HEKfectin (Bio-Rad, München, Germany). 5 × 10^5 ^HEK293T cells were seeded and cultivated overnight into six well tissue culture plates (Sarstedt, Nürnbrecht, Germany) with 2 mL Dulbecco's Modified Eagle Medium (DMEM), supplemented with 2 mM L-glutamine, 1.5 g/L sodium bicarbonate and 4.5 g/L glucose, 8% (v/v) fetal calf serum (FCS) and 1% (w/v) penicillin/streptomycin (PAA, Parsing, Austria) at 37°C in 7% CO_2 _atmosphere and at 95% humidity. For the transfection 2.5 μg of plasmid DNA, encoding the scFv-Fc gene construct and 10 μL HEKfectin were preincubated in DMEM before the DNA-liposome complexes were then added to the HEK cells. The cells were incubated overnight with the transfection mixture before the medium was exchanged to fresh one on the following day. After 72 hours culture supernatants containing the scFv-Fc fusion proteins were harvested. The scFv-Fc fusion protein content of the collected supernatants was analyzed using a human IgG capture ELISA as previously described [57].

## Authors' contributions

MIK, BH performed the most experiments and helped to draft the manuscript. CN and TR performed some of the experiments. In addition, BH participated in the design and coordination of the study. TS and HJM particpated in the design and coordination of the study and helped to draft the manuscript. MH drafted the manuscript, participated in the design and coordination of the study and performed some of the experiments. SD conceived the project and wrote the grant application, particpated in the design and coordination of the study and helped to draft the manuscript. All authors read and approved the final manuscript.
